# Biophotonic sensors with integrated Si_3_N_4_-organic hybrid (SiNOH) lasers for point-of-care diagnostics

**DOI:** 10.1038/s41377-021-00486-w

**Published:** 2021-03-26

**Authors:** Daria Kohler, Gregor Schindler, Lothar Hahn, Johannes Milvich, Andreas Hofmann, Kerstin Länge, Wolfgang Freude, Christian Koos

**Affiliations:** 1 Institute of Photonics and Quantum Electronics (IPQ), Karlsruhe Institute of Technology (KIT), Engesserstrasse 5, 76131 Karlsruhe, Germany; 2 Institute of Microstructure Technology (IMT), Karlsruhe Institute of Technology (KIT), Hermann-von-Helmholtz-Platz 1, 76344 Eggenstein-Leopoldshafen, Germany; 3 Robert Bosch GmbH, Robert-Bosch-Campus 1, 71272 Renningen, Germany; 4 Institute for Automation and Applied Informatics (IAI), Karlsruhe Institute of Technology (KIT), Hermann-von-Helmholtz-Platz 1, 76344 Eggenstein-Leopoldshafen, Germany

**Keywords:** Integrated optics, Biophotonics, Optical sensors, Lasers, LEDs and light sources, Silicon photonics

## Abstract

Early and efficient disease diagnosis with low-cost point-of-care devices is gaining importance for personalized medicine and public health protection. Within this context, waveguide-(WG)-based optical biosensors on the silicon-nitride (Si_3_N_4_) platform represent a particularly promising option, offering highly sensitive detection of indicative biomarkers in multiplexed sensor arrays operated by light in the visible-wavelength range. However, while passive Si_3_N_4_-based photonic circuits lend themselves to highly scalable mass production, the integration of low-cost light sources remains a challenge. In this paper, we demonstrate optical biosensors that combine Si_3_N_4_ sensor circuits with hybrid on-chip organic lasers. These Si_3_N_4_-organic hybrid (SiNOH) lasers rely on a dye-doped cladding material that are deposited on top of a passive WG and that are optically pumped by an external light source. Fabrication of the devices is simple: The underlying Si_3_N_4_ WGs are structured in a single lithography step, and the organic gain medium is subsequently applied by dispensing, spin-coating, or ink-jet printing processes. A highly parallel read-out of the optical sensor signals is accomplished with a simple camera. In our proof-of-concept experiment, we demonstrate the viability of the approach by detecting different concentrations of fibrinogen in phosphate-buffered saline solutions with a sensor-length (*L-*)-related sensitivity of *S/L* = 0.16 rad nM^−1^ mm^−1^. To our knowledge, this is the first demonstration of an integrated optical circuit driven by a co-integrated low-cost organic light source. We expect that the versatility of the device concept, the simple operation principle, and the compatibility with cost-efficient mass production will make the concept a highly attractive option for applications in biophotonics and point-of-care diagnostics.

## Optical biosensors: light source integration

Optical sensors that comprise integrated lasers and that are compatible with cost-efficient mass manufacturing could be promising for point-of-care diagnostics. Daria Kohler and coworkers at Karlsruhe Institute of Technology (KIT) and Robert Bosch GmbH in Germany have now demonstrated biosensors that combine silicon-nitride (Si_3_N_4_) waveguides with co-integrated Si_3_N_4_-organic hybrid (SiNOH) lasers. The sensor consists of three main integrated parts: The optically-pumped SiNOH laser, a Mach-Zehnder interferometer acting as a biosensor, and a set of grating couplers for directing the output to a CCD camera. The SiNOH laser relies on a spiral Si_3_N_4_ waveguide, which is covered by a dye-doped organic cladding to provide optical gain. The devices are technically simple and can be efficiently realized by wafer-level printing techniques.

## Introduction

Integrated sensors based on optical waveguides (WGs) exhibit an enormous application potential in biophotonics and medical diagnostics, especially when it comes to multiplexed, highly sensitive detection of a wide variety of target molecules^1,2^. In bio-photonic applications, the visible (VIS, *λ* = 400, …, 700 nm) and short-wavelength near-infrared (NIR, *λ* = 700, …, 1100 nm) spectral ranges are of particular interest^3^, offering a low absorption^4^ and hence permitting large interaction lengths of the guided light with analytes in mostly aqueous solutions^5,6^. Within this context, silicon-nitride (Si_3_N_4_) has emerged as a powerful integration platform for WG-based sensor systems^7^. Its advantages are the low propagation loss over the wide spectral range between 400 nm and 2.3 µm^8–11^ in addition to the high refractive-index contrast between the Si_3_N_4_ WG core ($n_{{\mathrm{Si}}_3{\mathrm{N}}_4} = 2.01$ @ *λ* = 600 nm^12^) and the silicon dioxide cladding ($n_{{\mathrm{SiO}}_2} = 1.46$ @ *λ* = 600 nm^13^). Moreover, Si_3_N_4_-based photonic integrated circuits (PICs) can be efficiently fabricated in large quantities using mature wafer-scale processes that offer high yield and are that accessible through a worldwide ecosystem of photonic foundries^8,9^. This opens a path towards cost-efficient mass production of highly functional sensor chips for one-time use in point-of-care diagnostics.

However, with a few exceptions in the mid-infrared wavelength range^14^, biosensors on the Si_3_N_4_ integration platform remain limited to mainly passive circuits and usually rely on external light sources coupled to the chip^5,15–17^. This requires delicate fibre-chip coupling schemes that are subject to stringent mechanical tolerances, which conflicts with the demand for technically simple low-cost sensor systems for point-of-care diagnostics. Cointegration of sensor circuits with on-chip lasers might represent an alternative, but all demonstrations have so far been limited to NIR sources that are first realized on a separate substrate and that are then flip-chip mounted onto passive Si_3_N_4_ PICs in a dedicated assembly step^18^. This involves serial assembly processes and thus mitigates most of the scalability advantages of highly parallel wafer-level mass fabrication. It is hence uncertain whether high-precision assembly of discrete laser dies could comply with the stringent cost limitations of disposable biosensors.

In this paper, we demonstrate a Si_3_N_4_ biosensor monolithically co-integrated with a low-cost hybrid laser source operating at visible wavelengths. The laser relies on the concept of Si_3_N_4_-organic hybrid (SiNOH) integration and combines passive Si_3_N_4_ WG cores with dye-doped organic cladding materials^19–23^. The devices are technically simple and can be efficiently realized by wafer-level printing techniques. SiNOH lasers may either be optically pumped by an external laser or a light-emitting diode (LED) without any high-precision alignment of the pump spot or physical contact with the chip. In a proof-of-concept experiment, we demonstrate the viability of the sensor system by detecting different concentrations of fibrinogen in an aqueous buffer solution. To our knowledge, this is the first demonstration of a Si_3_N_4_ photonic integrated circuit driven by an on-chip laser source emitting at visible wavelengths.

## Results

### Concept

**Fig. 1:**
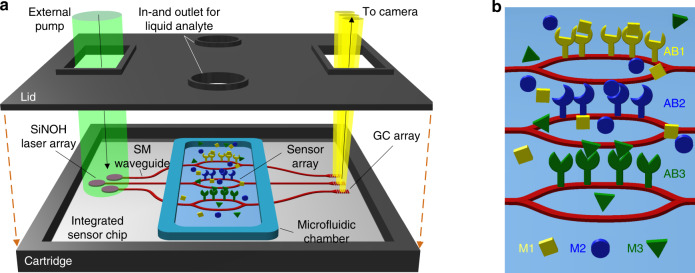
Concept of the Si_3_N_4_-based biosensor with co-integrated lasers. **a** The Si_3_N_4_ chip combines a SiNOH laser array with an array of sensors, illustrated as Mach-Zehnder interferometers (MZI), and is placed in a cartridge (black) containing windows for optical pumping and read-out. The sensor comprises a microfluidic chamber with an in- and outlet for the liquid analyte solution, which is formed by the chip surface, the cartridge lid, and an elastic seal (blue) between the chip surface and lid acting as the chamber sidewalls. The integrated SiNOH lasers are pumped by an external light source with a large pump spot size such that high-precision alignment is not needed. The light originating from the sensor output is radiated from the chip to a read-out camera by grating couplers. **b** Simplified schematic of the MZI-based sensor. Each MZI contains one sensing and one reference arm. The sensing arms are functionalized with individual antibodies AB1, AB2, and AB3 that bind specific target molecules M1, M2, and M3, respectively, to the WG surfaces

The concept of a Si_3_N_4_-based biosensor with co-integrated SiNOH lasers is shown in Fig. 1a. For robust handling purposes in point-of-care applications, the sensor chip is placed in a cartridge (black) containing windows for optical pumping and read-out. The sensor comprises a microfluidic chamber with in- and outlets for the liquid analyte solution. The chamber is formed by the chip surface, the cartridge lid, and an elastic seal (blue) between the chip surface and the lid. The SiNOH lasers are pumped from the top by an external light source with a large spot size such that high-precision alignment of the chip with respect to the pump beam is not needed. The generated laser light is coupled with a Si_3_N_4_ single-mode WG and guided to an array of on-chip sensors. The output light is radiated through the read-out window by grating couplers and captured by a camera. Optical pumping and read-out offer the advantage that no physical contact with the chip is required and thus greatly simplifies the handling of the devices—a key aspect in point-of-care applications. Figure 1b shows a simplified schematic of the sensor section, consisting of three Mach-Zehnder interferometers (MZIs) with one sensing arm and one reference arm each. The sensing arms are functionalized with individual antibodies AB1, AB2, and AB3 that bind specific target molecules M1, M2, and M3, respectively, to the WG surface. The reference arms are not functionalized.

### Proof-of-concept system

**Fig. 2:**
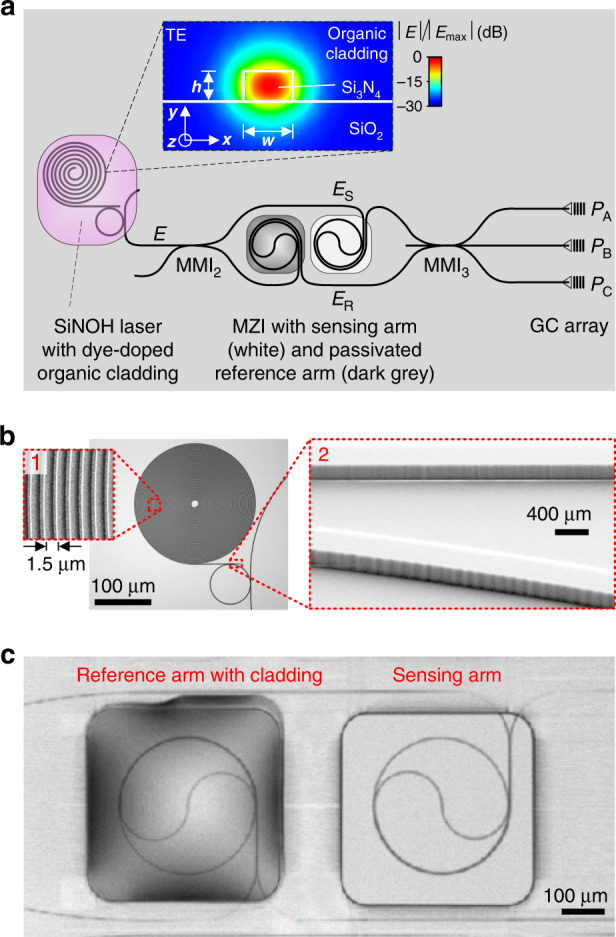
Fabricated demonstrator chip, combining a SiNOH laser with an MZI-based biosensor. **a** The SiNOH laser cavity is an open-ended spiral WG that is evanescently coupled to a ring resonator, which feeds the input WG of the MZI-based sensor circuit. Resonant coupling of the counter-propagating modes in the ring leads to narrowband frequency-selective back-reflection from the outer end of the spiral. Broadband optical feedback from the inner end of the spiral is provided by reflection from the open WG end at the centre in combination with roughness-induced backscattering along the 20-mm-long spiral WG. The cladding window of the SiNOH laser (magenta) is filled with dye-doped PMMA acting as an organic gain material. The inset shows a typical cross-section (width *w* and height *h*) of a SiNOH WG along with the the simulated electric field magnitudes $| {\overrightarrow E } |$ of the fundamental quasi-TE mode. The sensor MZI is formed by a 2 × 2 MMI splitter (MMI_2_) and a 3 × 3 MMI combiner (MMI_3_), of which only two input ports are used. The splitter distributes the light originating from the SiNOH laser equally to the two spiral-shaped arms. The sensing arm (white) is exposed to the analyte solution, while the reference arm (dark grey) is passivated by a cover material. The three output signals of MMI_3_ are coupled from the chip by a GC array and recorded by a camera (not shown). **b** Light-microscopy image of a SiNOH laser cavity without cladding. The spiral is densely coiled up with a 1.5-µm distance between neighbouring WGs, see Inset 1. The waveguides are subject to a certain sidewall roughness in the form of vertical grooves, see Inset 2. This roughness may lead to loss and to a resonantly enhanced coupling of the counter-propagating modes in the ring resonator. **c** Spiral-shaped WG in the MZI arms. For passivation purposes, the reference arm is covered with a low-index glue that is approximately index-matched to the aqueous analyte solution applied to the sensing arm

To demonstrate the viability of the sensor concept shown in Fig. 1, we realized a proof-of-principle demonstrator that combines an on-chip SiNOH laser with an MZI-based biosensor in a microfluidic chamber. The layout of the underlying Si_3_N_4_ chip is shown in Fig. 2a. The MZI is placed between the SiNOH laser and the grating coupler (GC) array. The laser is sufficiently far from the GC array to avoid any optical cross-talk by stray light originating from the pump laser.

The SiNOH laser relies on a passive Si_3_N_4_ WG, which is embedded into a light-emitting cladding that can be optically pumped (Inset of Fig. 2a). Details of the SiNOH laser have been presented in an earlier publication^20^. In our experiments, the cladding consisted of 800-nm-thick polymethylmethacrylate (PMMA) doped with an organic dye. This cladding can be deposited via simple wafer-level dispensing or ink-jet printing processes. The SiNOH laser cavity is realized as an open-ended spiral-shaped Si_3_N_4_ WG with a distance of 1.5 µm between neighbouring turns (Fig. 2b). The spiral WG is evanescently coupled to a ring resonator, where the resonant coupling of counter-propagating modes leads to frequency-selective optical feedback. The ring resonator also feeds the output WG, thus yielding an MZI-based sensor circuit. The overall footprint of the SiNOH laser amounts to less than 0.3 × 0.3 mm²—much smaller than the microfluidic structures required for the handling of the analyte, which typically extend over several millimetres. Note that the concept of combining passive inorganic WG structures with functional organic cladding materials has previously been exploited in silicon photonic circuits operated in the near-infrared wavelength range. This silicon-organic hybrid (SOH) integration concept lends itself to electro-optic modulators with a record-high efficiency^24^ and to low-cost on-chip laser sources^25^.

The sensor circuit itself consists of a 2 × 2 multimode interference coupler (MMI_2_) that splits the incoming light into the two spiral-shaped arms of the MZI (Fig. 2a, c). The sensing arm (white) is exposed to the analyte, while the reference arm (dark grey) is covered by a protective layer (Fig. 2c). The sensing and reference arms exhibit the same geometrical length, i.e., *L* = 1.8 mm. The output fields of the sensing arm (${\underline{E}} _{\mathrm{S}}$) and the reference arm (${\underline{E}} _{\mathrm{R}}$) are fed to the two input ports of a 3 × 3 MMI (MMI_3_). The three output signals propagate to a GC array, which radiates the light to the top. The MMI and GC are designed for operation under quasi-TE polarization, for which the horizontal component (*x*-component) of the optical mode field dominates (please refer to the inset of Fig. 2a).

### On-chip SiNOH laser

The SiNOH laser design is slightly different from that of the devices presented in our former publications^19,20^. Specifically, we found that due to the high gain provided by the dye-doped organic cladding material, lasing was possible even for cavities with fairly low Q-factors. We exploit this fact by replacing the closed-spiral cavity used in ref. ^20^ with an open spiral, which does not require a large S-turn at the centre and hence allows us to greatly increase the overlap with the Gaussian pump spot. This becomes more obvious when comparing the area fill factor of the open SiNOH laser spiral shown in Fig. 2b to the sensor spirals in the MZI arms shown in Fig. 2c. The open laser spiral is densely coiled with a distance of 1.5 µm between the neighbouring WGs and fills a circular area with a 100-µm radius. It is pumped with a Gaussian spot (green) exhibiting a full-width half-maximum of 160 µm. The slightly multimoded (MM) spiral WG reveals a width of 500 nm that is tapered down to 300 nm to allow single-mode coupling to a ring resonator with a radius of 40 µm and a free spectral range of FSR = 0.66 nm (540 GHz). To avoid gain competition between the various lasing modes, the FSR was deliberately chosen to slightly exceed the bandwidth of the homogeneous gain broadening that was previously estimated to be on the order of 0.6 nm^20^. It should be noted that the FSR must not be chosen unnecessarily large to ensure that all dye molecules emit into a spectrally sufficiently close lasing mode. This leads to polychromatic laser emission—a feature that must be considered when designing the sensor coupled to the SiNOH laser. Within the ring, resonantly enhanced coupling of the counter-propagating modes leads to single-mode frequency-selective reflection of light back into the spiral. This coupling arises from the surface roughness of the WG ring (as shown in Fig. 2b). Moreover, broadband optical feedback is provided from the inner end of the spiral through reflection from the open WG end at the centre, possibly in combination with the roughness-induced backscattering along the 18-mm-long spiral WG. We also measured the propagation loss of the Si_3_N_4_ WG in the SiNOH cavity, which, for an undoped PMMA cladding, amounted to approximately 5 dB/cm for the 500-nm-wide multimode spiral WG and to approximately 7 dB/cm for the narrower 300-nm-wide single-mode WG. A more detailed discussion of the optical feedback in the SiNOH laser cavity has been provided in Supplementary Note 1.

**Fig. 3:**
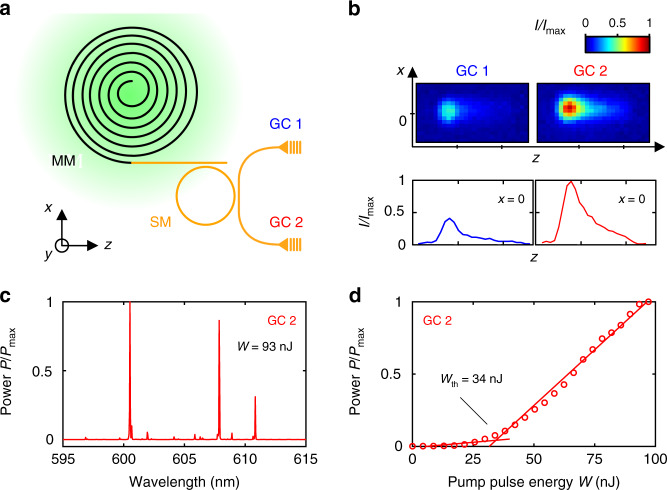
Characterization of SiNOH laser. **a** SiNOH laser geometry: a multimode WG (MM, black, w = 500 nm) embedded in the gain medium is coiled into a spiral with an outer diameter of 200 µm.The arrangement offers a large overlap of the spiral WG with the Gaussian pump spot exhibiting a full-width half-maximum of 160 µm (green). The MM WG is tapered down to a single-mode (SM) WG subject to *w* = 300 nm (orange), which is evanescently coupled to a SM ring resonator (*Q* = 5 × 10^3^). The ring resonator is coupled to the SM output WG that guides the light to GC1 and GC2, which are both optimized for quasi-TE polarization. **b** Colour-coded intensity of the SiNOH laser light emitted from GC1 and GC2 (upper row) along with a cross-section of the emission profile along the line of *x* = 0 (bottom row). The emission from the GC is recorded by a CCD camera (three-fold magnification) and normalized to the maximum detected power intensity *I*_max_ measured at GC2. By comparing these plots, we find that the clockwise-propagating power in the ring is three times as high as the counter-clockwise-propagating power, which provides feedback into the pumped spiral. **c** Normalized emission spectrum of the SiNOH laser recorded under quasi-TE-polarization at GC2 (resolution bandwidth: 60 pm; integration time: 2 s). Three dominant lasing modes are shown. **d** Normalized laser output power as a function of the pump-pulse energy *W*. We find a lasing threshold of *W*_th_ = 34 nJ

In the first step, we characterize the SiNOH lasers without attached biosensors, see Fig. 3. Figure 3a shows the associated test structure with two output grating couplers GC1 and GC2. The device is optically pumped by a frequency-doubled Nd:YLF pulsed laser (CL523, CrystaLaser, Reno, USA) with an emission wavelength of 523 nm, a pulse duration of 20 ns, and a repetition rate of 20 Hz. Within the single-mode (SM) ring resonator, the clockwise-propagating mode is predominantly excited, but it also experiences resonantly enhanced coupling to the counter-clockwise-propagating mode because of structural imperfections such as sidewall, see Inset 2 of Fig. 2b. This is confirmed by comparing the output power found at GC1 and GC2, see Fig. 3b. The top row shows the colour-coded intensity distribution of the light radiated from GC1 and GC2 as recorded by a camera. The bottom row shows cross-sectional intensity plots along the main axes of the grating couplers, i.e. at *x* = 0. The emission from GC2 is approximately three times as high as the emission from GC1, indicating that the clockwise-propagation mode is predominantly excited but experiences substantial coupling to its clockwise-propagating counterpart.

For a further investigation, we captured the light from GC2 with a single-mode fibre and connected it to a spectrometer with a resolution bandwidth of 60 pm and an integration time of 2 s. Figure 3c shows the emission spectrum at a pump-pulse energy of *W* = 93 nJ. The spectrum is normalized to the highest peak and shows three dominant quasi-TE-polarized lines within the recorded spectral range of 10 nm. The peak power of the emitted laser pulse is on the order of 100 mW, which is twice as high as that of previously demonstrated SiNOH lasers^20^. This improvement was achieved by using open-spiral cavities, which do not require a large S-turn at the centre and thus provide a better overlap with the pump spot than their closed-spiral counterparts used in our previous demonstration. The normalized average output power *P*/*P*_max_ vs. the pump-pulse energy *W* is shown in Fig. 3d. At each pump-pulse energy level, the output power is measured by integrating the spectrum, as shown in Fig. 3c, overall wavelengths. We find a clear lasing threshold with a threshold pump-pulse energy of *W*_th_ = 34 nJ. We also investigated the output power emitted from GC1 as a function of the pump-pulse energy, observing that the lasing threshold is essentially the same as that extracted from GC2, see Supplementary Fig. S1. This confirms the notion that the emission at GC1 results from resonant coupling of counter-propagating modes in the ring. By investigating the output spectra of GC2 for different pump-pulse energies, we find that the three lasing modes exhibit distinct thresholds with no indication of gain competition, see Supplementary Fig. S2. Further details on the SiNOH spiral laser and the emission characteristics are contained in Supplementary Note 1.

### MZI-based sensors and polychromatic laser source

MZI-based sensors offer the general advantage that they are robust with respect to mechanical stress and temperature fluctuations, in particular when the sensing and reference arm rely on WGs which feature identical or very similar cross-sections and which are placed in close vicinity to each other. Temperature fluctuations in the chip will then affect both MZI arms in the same way, without any impact on the measurement result, which only depends on the phase difference at the output of the two arms. In general, 2 × 1 MMI, 2 × 2 MMI, or 3 × 3 MMI may be used as power combiners in MZI-based biosensors. MZI based on 2 × 2 MMI and 3 × 3 MMI allow for correction of laser-power fluctuations, which affects all output signals equally. This is in contrast to structures involving 2 × 1 MMI, for which a phase shift in the sensor arm is indistinguishable from a power fluctuation in the light source. Compared to 2 × 2 MMI, 3 × 3 MMI offers several additional sensing advantages. First, the difference Δ*ϕ* of the phase shifts in the two MZI arms can be directly reconstructed from the power levels *P*_A_, *P*_B_, and *P*_C_ measured at the three output ports A, B, and C, respectively, without any phase ambiguities within the usual 2π-interval. Second, appropriate signal processing allows achieving a sensitivity that is independent of the MZI operating point. Moreover, fabrication inaccuracies and temperature influences causing amplitude and phase errors of the MMI can be corrected by digital signal processing (DSP). In the following paragraphs, these advantages are explained in more detail.

As discussed in the previous section, polychromatic emission is a distinct feature of SiNOH lasers that is closely related to the gain medium. This aspect must be considered when describing the interplay of the light source with the sensor. In the following, we assume a polychromatic source with *M* spectral lines with optical angular frequencies *ω*_*m*_ (*m* = 1, 2, …, *M*) and complex amplitudes ${\underline{\hat E}} _m$, see Supplementary Note 3 for additional details. We represent the optical power of each spectral line by the squared magnitude of the respective complex field amplitude ${\underline{\hat E}} _m$, and the total power is given as the sum of the powers of the individual components, $P_{{\mathrm{source}}} = \mathop {\sum}\limits_{m = 1}^M {\left| {{\underline{\hat E}} _m} \right|^2}$, see Supplementary Note 3, Eq. (S7). Within the Si_3_N_4_ MZI WG, we assume propagation along the *z*-direction with propagation constant *β*_*m*_. The resulting fields associated with an individual spectral component are hence represented by ${\underline{E}} _m = {\underline{\hat E}} _m\exp \left[ {{\mathop{\rm{j}}\nolimits} \left( {\omega _m\,t - \beta _mz} \right)} \right]$, with *z* being the propagation distance along the WG, and the overall complex field is the superposition of ${\underline{E}} = \mathop {\sum}\nolimits_{m = 1}^M {{\underline{E}} _m}$. The physical electric field is obtained by calculating the real part, i.e. $E\left( {{\boldsymbol{r}},t} \right) = {\mathrm{Re}}\left[ {{\underline{E}} \left( {{\boldsymbol{r}},t} \right)} \right]$.

At the input of the MZI, the electric field is split by the 2 × 2 MMI into equal portions in the sensing and reference WGs, see Supplementary Notes 2 and 3 for a detailed mathematical description of the MZI operated by monochromatic and polychromatic sources, respectively. Splitting of the field in the 2 × 2 MMI leads to a phase shift of −*π*/2 in the field in the reference arm with respect to the field in the sensing arm. For simplicity, we assume that the reference and measurement arms exhibit equal geometrical lengths *L*. The effective index of the reference arm is denoted as *n*_eR_ and is assumed to remain constant during the sensing experiments since the reference arm is completely embedded into a passivation layer to isolate the Si_3_N_4_-based WG from the analyte. Regarding the sensing arm, the effective index in the absence of any analyte molecules is denoted as *n*_eS_. If the sensor is exposed to the analyte, adsorption of target molecules leads to a change Δ*n*_eS_ in the effective refractive index. At the input of the 3 × 3 MMI combiner, the electric field ${\underline{E}} _{\mathrm{R}}$ in the reference arm can thus be written as

1 $$\begin{array}{c}{\underline{E}} _{\mathrm{R}} = \mathop {\sum}\limits_{m = 1}^M {\frac{{{\underline{\hat E}} _m}}{{\sqrt 2 }}\;{\mathop{\rm{e}}\nolimits} ^{{\mathrm{j}}\left( {\omega _mt \, + \, \phi _{{\mathrm{R,}}m} - \pi /2} \right)}} ,\\ \phi _{{\mathrm{R,}}m} = - \beta _{{\mathrm{R,}}m}L = - k_{0,m}n_{{\mathrm{e,R,}}m}L,\quad \quad k_{0,m} = \omega _m/c\end{array}$$

The electric field ${\underline{E}} _{\mathrm{S}}$ in the sensing arm is

2 $$\begin{array}{c}{\underline{E}} _{\mathrm{S}} = \mathop {\sum}\limits_{m = 1}^M {\frac{{{\underline{\hat E}} _m}}{{\sqrt 2 }}\;{\mathop{\rm{e}}\nolimits} ^{{\mathrm{j}}\left( {\omega _mt \, + \, \phi _{{\mathrm{S,}}m} \, + \, {\Delta}\phi _{{\mathrm{S,}}m}} \right)}} ,\\ \phi _{{\mathrm{S,}}m} = - \beta _{{\mathrm{S,}}m}L = - k_{0,m}n_{{\mathrm{e,S,}}m}L,\quad \\ {\Delta}\phi _{{\mathrm{S,}}m} = - {\Delta}\beta _{{\mathrm{S,}}m}L = - k_{0,m}{\Delta}n_{{\mathrm{e,S,}}m}L\end{array}$$

The 3 × 3 MMI combiner at the output of the MZI leads to a superposition of the fields ${\underline{E}}_{\mathrm{S}}$ and ${\underline{E}}_{\mathrm{R}}$ with realtive phase shifts of 2*π*/3, 0, and −2*π*/3 at the ports A, B, and C, respectively^26^. The associated output powers *P*_A_, *P*_B_, and *P*_C_, respectively, are detected at the respective GCs, Fig. 2a, analogue-to-digital converted, and numerically processed by applying a Clarke transform, see Supplementary Note 3 for a more detailed mathematical description. This leads to a correlation function, which we refer to as the Clarke field ${\underline{s}}_{{\mathrm{poly}}}$^27,28^,

3 $$\begin{array}{c}{\underline{s}} _{{\mathrm{poly}}} = {\underline{s}} _{\mathrm{r}} + {\mathrm{j}}\,{\underline{s}} _{\mathrm{i}} = 2\left\langle {{\underline{E}} _{\mathrm{S}}{\underline{E}} _{\mathrm{R}}^ \ast } \right\rangle _T\\ = \mathop {\sum}\limits_{m = 1}^M {\left[ {\left| {{\underline{\hat E}} _m} \right|^2{\mathop{\rm{e}}\nolimits} ^{{\mathrm{j}}\,\left[ {{\Delta}\phi _{{\mathrm{S,}}m} \, + \, \pi /2 \, + \, \left( {\phi _{{\mathrm{S,}}m} - \phi _{{\mathrm{R,}}m}} \right)} \right]}} \right]} \\ = 2P_{\mathrm{B}} - \left( {P_{\mathrm{C}} + P_{\mathrm{A}}} \right) + {\mathrm{j}}\sqrt 3 \left( {P_{\mathrm{C}} - P_{\mathrm{A}}} \right)\end{array}$$

**Fig. 4:**
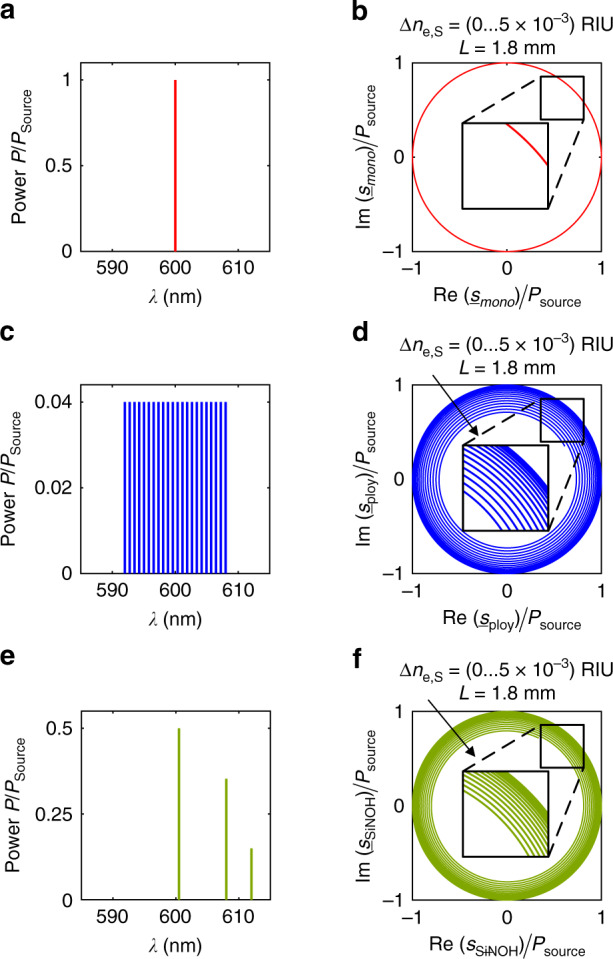
Calculated complex Clarke fields ${\underline{s}}$ of an MZI with 3 × 3 MMI combiner at the output for different light sources. **a**, **c**, **e** Normalized emission spectrum for a monochromatic laser line, Subfigure 4a, for a polychromatic laser with equally spaced lines of equal powers, Subfigure 4c, and for a SiNOH laser with three monochromatic lasing lines located at different wavelengths and exhibiting different powers, Subfigure 4e. **b**, **d**, **f** Clarke fields ${\underline{s}}$ calculated from the three output powers of the 3 × 3 MMI according to Eq. 3 and plotted in the complex ${\underline{s}}$-plane. The geometrical lengths of the sensing and reference arms amount to *L* = 1.8 mm, and the effective refractive-index difference Δ*n*_e,S_ varies between 0 and 5 × 10^–3^ RIU. We assume a wavelength of 600 nm. **b** For the monochromatic laser, Subfigure 4a, ${\underline{s}} _{{\mathrm{mono}}}$ describes a circle with radius $| {{\underline{s}} _{{\mathrm{mono}}}} | = P_{{\mathrm{Source}}}$, centred at the origin. **d** For the polychromatic laser, Subfigure 4c, the magnitude $| {s_{{\mathrm{poly}}}} |$ decreases with increasing Δ*n*_e,S,_ and ${\underline{s}} _{{\mathrm{poly}}}$ describes a spiral. **f** For the SiNOH laser described in Fig. 3, the magnitude $| {{\underline{s}} _{{\mathrm{SiNOH}}}} |$ decreases with increasing Δ*n*_e,S,_, as shown in Subfigure 4f, but the decrease is smaller than for the case of $| {{\underline{s}} _{{\mathrm{poly}}}} |$. In real experiments, non-ideal MMIs and GCs could lead to an elliptical deformation of the otherwise circular or spiral-shaped locus of the corresponding Clarke field ${\underline{s}}$ in the complex plane. This can be compensated by transforming the ellipse back into a circle with a unity radius^27,28^

For a monochromatic light source (*M* = 1), as shown in Fig. 4a, Eq. (3) can be simplified, see Supplementary Note 2,

4 $$\begin{array}{c}{\underline{s}} _{{\mathrm{mono}}} = {\underline{s}} _{\mathrm{r}} + {\mathop{\rm{j}}\nolimits} {\underline{s}} _{\mathrm{i}} = 2{\underline{E}} _{\mathrm{S}}{\underline{E}} _{\mathrm{R}}^ \ast = \left| {{\underline{\hat E}} } \right|^2{\mathop{\rm{e}}\nolimits} ^{{\mathrm{j}}\,\left[ {{\Delta}\phi _{\mathrm{S}} \, + \, \pi /2 \, + \, \left( {\phi _{\mathrm{S}} - \phi _{\mathrm{R}}} \right)} \right]},\\ \left| {{\underline{s}} _{{\mathrm{mono}}}} \right| = \left| {{\underline{\hat E}} } \right|^2 = P_{{\mathrm{source}}}\end{array}$$

Note that we have assumed a lossless sensor circuit to simplify our analysis. In case of a lossy device, the power *P*_Source_ in Eq. (4) has to be accordingly reduced, but this does not have any consequences for the subsequent considerations. In the monochromatic case, the phase shift between the sensing and reference arms can be determined from the total phase Δ*ϕ* of the complex Clarke field according to Eq. (4),

5 $$\begin{array}{c}{\Delta}\phi = {\Delta}\phi _{\mathrm{S}} + \pi /2 + \left( {\phi _{\mathrm{S}} - \phi _{\mathrm{R}}} \right)\\ = - \frac{\omega }{c}\left[ {{\Delta}n_{{\mathrm{e,S}}} + \left( {n_{{\mathrm{e,S}}} - n_{{\mathrm{e,R}}}} \right)} \right]L + \pi /2\end{array}$$

The phase Δ*ϕ* hence changes with Δ*n*_eS_. For a sufficiently slow continuous variation in the phase shift during a binding experiment, Δ*ϕ* can be extracted from the measured data by unwrapping the Clarke phase $\arg \left( {{\underline{s}} _{{\mathrm{mono}}}} \right)$,

6 $${\Delta}\phi = {\mathop{\rm{unwrap}}\nolimits} \left( {\arg \left( {{\underline{s}} _{{\mathrm{mono}}}} \right)} \right)$$

For polychromatic light sources, we find that a relationship similar to Eq. (6) can be adopted to extract the phase shift Δ*ϕ* from the associated Clarke field ${\underline{s}} _{{\mathrm{poly}}}$, provided that the maximum frequency span of the source is smaller than the free spectral range of the generally unbalanced MZI, see Supplementary Note 3 for details,

7 $$\left| {\omega _m - \omega _0} \right| \ll \frac{c}{{{\Delta}n_{{\mathrm{e,g}}}L}}\quad \forall \;m,\quad {\Delta}n_{{\mathrm{e,g}}} = {\Delta}n_{{\mathrm{e,g,S}}} + \left( {n_{{\mathrm{e,g,S}}} - n_{{\mathrm{e,g,R}}}} \right)$$

where the centre frequency *ω*_0_ is given by the average of all emission frequencies *ω*_*m*_, and $\omega _0 = \mathop {\sum}\nolimits_{m = 1}^M {\omega _m} /M$.

The sensor sensitivity is defined by the magnitude of the derivative of the complex Clarke field ${\underline{s}}$ with respect to the phase shift Δ*ϕ*:

8 $$S_{{\mathrm{Clarke}}} = \left| {\frac{{{\mathop{\rm{d}}\nolimits} {\underline{s}} }}{{{\mathop{\rm{d}}\nolimits} {\Delta}\phi }}} \right| = \left| {{\underline{s}} } \right|,\quad \quad {\underline{s}} \in \left\{ {{\underline{s}} _{{\mathrm{mono}}},{\underline{s}} _{{\mathrm{poly}}}} \right\}$$

For an ideal MZI operated by a monochromatic laser, the sensitivity remains constant for all phase shifts Δ*ϕ*, $S_{{\mathrm{Clarke}}} = \left| {{\mathop{\rm{d}}\nolimits} {\underline{s}} _{{\mathrm{mono}}}/{\mathop{\rm{d}}\nolimits} {\Delta}\phi } \right| = \left| {{\underline{\hat E}} } \right|^2$, and ${\underline{s}} _{{\mathrm{mono}}}$ describes a circle with radius $\left| {{\underline{s}} _{{\mathrm{mono}}}} \right| = \left| {{\underline{\hat E}} } \right|^2 = P_{{\mathrm{source}}}$ in the complex ${\underline{s}}$-plane when changing Δ*n*_eS_ and Δ*ϕ*, as shown in Fig. 4a, b, respectively. In contrast to this, real devices are subject to fabrication imperfections and temperature fluctuations, leading to deviations of the relative shifts at the three optical output ports of the 3 × 3 MMI from the ideal value of 2*π*/3. In addition, the GC efficiencies of the three output ports might differ from each other. These effects lead to an elliptical deformation of the locus of ${\underline{s}}$ in the complex plane and to a shift of the centre away from the origin ${\underline{s}} = 0$^27^. This error is generally corrected by fitting an ellipse to measured data and by transforming this ellipse back to a circle with a unity radius centred at the origin^27,28^. In the following analysis, we assume that this correction has been performed, and we thus consider the case of an ideal sensor circuit.

While the amplitude of the Clarke field $\left| {{\underline{s}} _{{\mathrm{mono}}}} \right|$ for a monochromatic laser line remains constant with increasing Δ*n*_eS_, the amplitude of the Clarke field $| {{\underline{s}} _{{\mathrm{poly}}}} |$ for polychromatic lines may depend on Δ*n*_eS_ ≈ Δ*n*_e,g,S_, see Supplementary Note 3, Eq. (S9). For simplicity, we assume a light source with $M \gg 1$ equidistant laser lines within a maximum spectral range Δ*ω*_max_, all exhibiting equal field amplitudes ${\underline{\hat E}} _m = {\underline{\hat E}}$, as shown in Fig. 4c. In this case, an approximation is possible; please refer to Supplementary Note 3, Eq. (S20),

9 $$\begin{array}{c}| {{\underline{s}} _{{\mathrm{poly}}}} | = 2\left| {{\underline{\hat E}} _{\mathrm{S}}{\underline{E}} _{\mathrm{R}}^ \ast } \right| = \left| {{\underline{\hat E}} } \right|^2M\frac{{\sin \left( {\frac{{{\Delta}n_{{\mathrm{e,g}}}L}}{{2c}}{\Delta}\omega _{\max }} \right)}}{{\frac{{{\Delta}n_{{\mathrm{e,g}}}L}}{{2c}}{\Delta}\omega _{\max }}}\\ = P_{{\mathrm{source}}}\frac{{\sin \left( {\frac{{{\Delta}n_{{\mathrm{e,g}}}L}}{{2c}}{\Delta}\omega _{\max }} \right)}}{{\frac{{{\Delta}n_{{\mathrm{e,g}}}L}}{{2c}}{\Delta}\omega _{\max }}}\end{array}$$

Equation (9) indicates that the magnitude $| {{\underline{s}} _{{\mathrm{poly}}}} |$ and hence the sensitivity $S_{{\mathrm{Clarke}}} =| {{\underline{s}} _{{\mathrm{poly}}}}|$ decrease with increasing Δ*n*_e,g_, especially for large sensor arm lengths *L*. Figure 4d shows the Clarke field ${\underline{s}} _{{\mathrm{poly}}}$ in the complex ${\underline{s}}$-plane for *M* = 25 monochromatic equidistant laser lines centred at 600 nm, which are separated by an FSR = 0.66 nm (540 GHz) and exhibit equal powers, see Fig. 4c. The geometrical length of the MZI arms amounts to *L* = 1.8 mm, and the effective group refractive-index Δ*n*_e,g,S_ varies between 0 and 5 × 10^−3^ RIU, leading to a phase shift Δ*ϕ* between 0 and 100 rad.

We also perform a sensitivity analysis using the specific spectral characteristics measured for our SiNOH lasers. For these devices, we expect a lasing spectrum with several monochromatic lines exhibiting different powers within a maximal spectral range of Δ*ω*_max_ = 2*π* × 10 THz (Δ*λ* = 15 nm)^20^. As an example, Fig. 3c shows the spectrum of three dominant lines in the spectral range of Δ*λ* = 10 nm, which is approximated by three monochromatic lines in Fig. 4e. The electric fields are calculated by using Eqs. (S8) and (S9) in Supplementary Note 3. Equation (S3) in Supplementary Note 2 and Eq. (S10) in Supplementary Note 3 can then be used to calculate the corresponding complex Clarke field ${\underline{s}} _{{\mathrm{SiNOH}}}$. Compared to $| {{\underline{s}} _{{\mathrm{poly}}}} |$, Fig. 4d, the magnitude $\left| {{\underline{s}} _{{\mathrm{SiNOH}}}} \right|$ of the Clarke field decreases less with increasing Δ*n*_e,g_, as shown in Fig. 4f, and the same is true for the sensitivity.

In the experiment described in the next section, we adopt an MZI with a sensor arm length of *L* = 1.8 mm. For a worst-case estimate of the sensitivity degradation, we assume a polychromatic source as in Fig. 4c and Eq. (9) with equidistant spectral lines spread over a spectral range of 15 nm. This leads to a minimum amplitude of the Clarke field $| {{\underline{s}} _{{\mathrm{poly}}}} | = 0.85\left| {{\underline{s}} _{{\mathrm{mono}}}} \right|$, i.e. a maximum degradation of the sensitivity of 15%. We hence conclude that for mm-scale sensor lengths, the polychromatic emission of SiNOH lasers should not lead to a notable degradation of the sensor performance.

### Experimental sensor-system demonstration

**Fig. 5:**
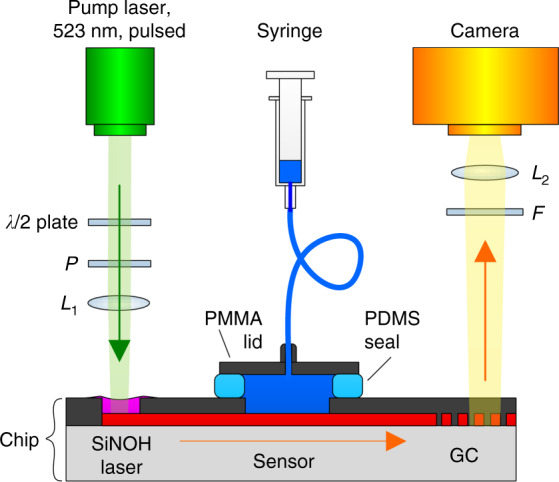
Schematic of the experimental setup. The SiNOH laser is pumped with a free-space beam from a pulsed laser, which is focused by lens *L*_1_. The pulse energy is varied by rotating a half-wave (*λ*/2) plate with respect to a fixed linear polarizer *P*. The SiNOH laser is coupled to an on-chip WG (red) that guides the light to the interferometric sensor, please refer to Fig. 2a for a top view of the sensor chip. At the sensor output, light is radiated to an approximately surface-normal direction of the chip by a GC array. A long-pass filter *F* is used to suppress any stray light from the pump laser. A subsequent lens *L*_2_ focuses the light on a CCD camera. The liquid analyte is pumped by a syringe through the fluidic chamber that is formed by the chip surface and the PMMA lid with a PDMS seal in between. The volumetric flow rate is 0.6 mL s^−1^

To demonstrate the viability of sensor systems based on SiNOH light sources, we perform a proof-of-concept experiment, see Fig. 5 for the associated setup. Pump light generated by a frequency-doubled Nd:YLF pulsed laser (CL523, CrystaLaser) with an emission wavelength of 523 nm, a pulse duration of 20 ns, and a repetition rate of 20 Hz is focused on the SiNOH laser by a lens *L*_1_. The pulse energy is varied by a half-wave (*λ*/2) and a subsequent polarizer *P*. The SiNOH laser is coupled to an on-chip WG that guides the light to the MZI-based interferometric sensor, which contains a 3 × 3 MMI at the output, see Fig. 2a. At the output of the MMI, the light is radiated to the top by an array of GCs. A long-pass filter *F* suppresses any stray light from the pump laser, and a subsequent lens *L*_2_ focuses the light on a CCD camera (Stingray, Allied Vision) with a 3-fold magnification. Images are continuously recorded at an exposure time of *T* = 2 s. The intensity radiated by each GC is detected by summing the grey-scale values of a 30 × 40-pixel area around the corresponding intensity maximum of the camera. In the sensing experiment, the liquid analyte solution is pumped by a syringe through the fluidic chamber, which is formed by the chip surface and the PMMA lid with a PDMS seal in between. To attain a constant volumetric flow rate, the syringe is emptied using a stepper motor. In the subsequent sensing experiments, the SiNOH laser is driven with a pump-pulse energy of *W* = 500 nJ. The sensor arm has a length of *L* = 1.8 mm.

**Fig. 6:**
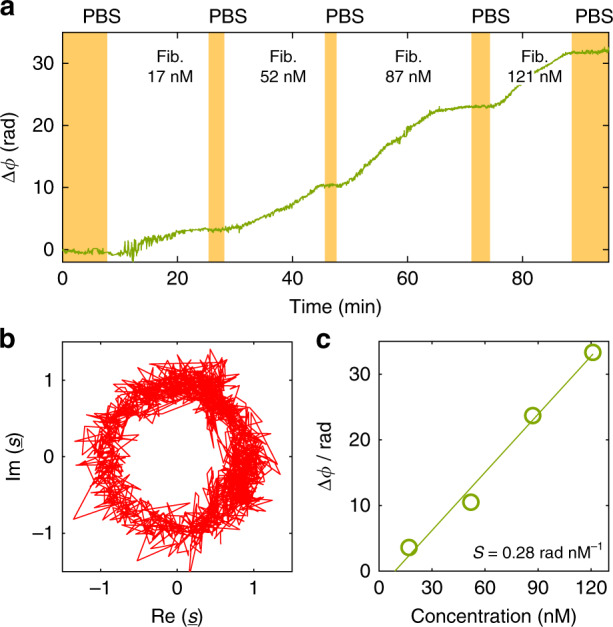
Demonstration of a sensor with SiNOH lasers as light sources, used for detection of fibrinogen dissolved in a phosphate-buffered saline (PBS) solution. **a** Binding curve, indicating the measured phase shift Δ*ϕ* as a function of time. During the time intervals marked in white, PBS solutions with fibrinogen concentrations of 17, 52, 87, and 121 nM are injected into the fluidic chamber. After each injection of a certain solution, pure PBS is used to flush the chamber during the time intervals indicated in yellow. For each concentration, the phase converges to a constant value when binding equilibrium is reached. **b** Locus-corrected complex Clarke field ${\underline{s}}$ for the various detection times in the complex *s*-plane. More details of the locus correction are provided in Supplementary Note 4. **c** Phase shift Δ*ϕ* vs. fibrinogen concentration: The phase shift for each concentration is calculated from the average phase measured during the subsequent PBS flushing period. As a reference Δ*ϕ* = 0 for the phase shift, we use the average phase measured during an initial PBS flushing period (marked in yellow) in the first eight minutes of the experiment. The phase shift Δ*ϕ* increases linearly with the concentration, leading to a sensitivity of *S* = 0.28 rad/nM

For the proof-of-concept demonstration, we detected the adsorption of fibrinogen onto the surface of the Si_3_N_4_ WG. This rather simple binding experiment allows us to investigate the performance of the sensor system without any impairment caused by complex media or complicated binding mechanisms. For our experiment, we prepared mixtures of four different concentrations (17, 52, 87, and 121 nM) of fibrinogen in phosphate-buffered saline solution (PBS). We then pumped these solutions sequentially through the fluidic chamber at a volumetric flow rate of 0.6 mL s^−1^, starting with the lowest concentration of 17 nM and ending with the highest concentration of 121 nM. Figure 6a shows the measured binding curve (green line), i.e. the detected phase shift Δ*ϕ* as a function of time. The white areas mark the periods during which the different fibrinogen solutions were pumped through the chamber. At the beginning of the experiment and after each fibrinogen injection, the chamber was flushed with PBS for several minutes, as indicated by the yellow areas in Fig. 6a. PBS flushing removed any unbound molecules from the WG surface.

Before calculating the Clarke fields, the raw power levels emitted from the different GCs were extracted from the recorded images by summing the grey-scale values of the respective 30 × 40-pixel areas as described above. To extract the phase shift Δ*ϕ*, we first removed the background offset of the three signals such that the resulting power levels $\tilde P_{\mathrm{A}}$, $\tilde P_{\mathrm{B}}$, and $\tilde P_{\mathrm{C}}$ were zero for destructive interference at ports A, B, and C, respectively. In the next step, the three port powers are normalized to the total power $\tilde P = \tilde P_{\mathrm{A}} + \tilde P_{\mathrm{B}} + \tilde P_{\mathrm{C}}$ measured at the respective time instant,

10 $$P_{\mathrm{X}} = \frac{{\tilde P_{\mathrm{X}}}}{{\tilde P_{\mathrm{A}} + \tilde P_{\mathrm{B}} + \tilde P_{\mathrm{C}}}},\quad X \in \left\{ {A,B,C} \right\}$$

The normalized dimensionless power quantities *P*_A_, *P*_B_, and *P*_C_ are then used to calculate the Clarke field according to Eq. (3). Note that the power normalization according to Eq. (10) removes the impact of laser-power fluctuations, which affect the numerator and the denominator in the same way. The phase shift, which is calculated from the normalized power quantities according to Eq. (3), should thus be robust with respect to laser intensity noise. The Clarke field obtained from Eq. (3) is then corrected by fitting a centred ellipse, see Section 3C, which is then transformed to a unity-radius circle^27,28^. Figure 6b shows the locus-corrected Clarke field ${\underline{s}}$ for the various detection times in the complex ${\underline{s}}$-plane. To extract the binding curve, we calculate the phase shift Δ*ϕ* by unwrapping the time-dependent phase of the Clarke field ${\underline{s}}$ in analogy to Eq. (6). Figure 6a shows the phase shifts measured over the duration of the experiment. Note that for the MZI-based sensors used in this experiment, the MMI couplers were already optimized for uniform power splitting ratios and phase shifts, such that the impact of the locus correction is rather small, see Supplementary Note 4 for a more detailed discussion. It should also be noted that the temporal evolution of the binding curve shown in Fig. 6a is dictated by the binding dynamics of the fibrinogen to the WG surface, in particular by the mobility of the target molecules in the analyte solution and by the binding affinity to the sensor surface, while camera-based acquisition system would have allowed to track much faster changes in the phase shift.

We further investigated the sensitivity of our sensor. As a reference, we use the average phase shift measured during the first 8 min of the experiment, in which the sensor is exposed to the PBS only. This reference phase is subtracted from all subsequently measured phase values. At each concentration, the flow of fibrinogen solution is maintained until equilibrium is reached, indicated by a steady value of the phase shift. Our observation of an equilibrium is in line with previous experiments of the concentration-dependent binding of fibrinogen onto silicon-nitride surfaces^29^. Note that the fibrinogen remains on the WG surface, even when flushing the chamber with the PBS. This observation cannot be explained by the rather simple binding model according to Langmuir^30^ but is in line with the experimental observations in ref. ^29^. We attribute this phenomenon to the high adsorption affinity of fibrinogen to the surface of the Si_3_N_4_ WG. Figure 6c shows the average phase shifts measured during PBS flushing after each fibrinogen injection interval as a function of the fibrinogen concentration. From the linear increase of the phase shift Δ*ϕ* with increasing concentration Δ*C*, we calculate a sensitivity of *S* = 0.28 rad nM^−1^. For the 1.8-mm-long sensor arm, this leads to a length-related sensitivity of *S/L* = Δ*ϕ*/(*L*Δ*C*) = 0.16 rad nM^−1^ mm^−1^. Based on the standard deviation *σ*_Δ*ϕ*_ = 0.2 rad of the binding curve when the sensor is exposed to the PBS only, Supplementary Note 4, we calculate the limit of detection to be LoD = 3*σ*_Δ*ϕ*_/*S* = 2.14 nM.

To our knowledge, our experiments represent the first demonstration of a WG-based sensor circuit on the Si_3_N_4_ platform driven by an on-chip laser source emitting at visible wavelengths. Previously demonstrated Si_3_N_4_-based sensor circuits with co-integrated light sources were either limited to mid-infrared wavelengths emitted by epitaxially grown quantum cascade lasers^14^ or relied on external light sources such as vertical-cavity surface-emitting lasers (VCSELs) that emit at near-infrared wavelengths and that are flip-chip mounted to the underlying Si_3_N_4_ substrate^18^. In comparison to these approaches, the SiNOH concept stands out due to its amenability to highly scalable mass fabrication by wafer-level printing of organic dye materials onto passive Si_3_N_4_ photonic integrated circuits.

## Discussion

Our proof-of-concept experiment demonstrates the viability of optical biosensors driven by co-integrated SiNOH lasers as highly scalable low-cost light sources, which can be adapted to a wide range of emission wavelengths by varying the gain material. Nevertheless, the concept leaves much room for further improvement for highly sensitive detection in point-of-care diagnostics. A short discussion of these aspects is provided in the following sections.

### Sensitivity limitations and improvements

In the fibrinogen sensing experiment presented in this work, we estimate a detection limit of LoD = 2.14 nM. Note, however, that this concentration-related detection limit is largely dictated by the specific binding experiment, in particular by the molecular mass of the analyte and by its binding affinity to the WG surface, such that a comparison to other sensing experiments is difficult. To benchmark the sensitivity of our sensor system with respect to the state of the art, we also estimate the limit of detection for homogeneous sensing LoD_hom_, i.e. for detection of a global refractive-index change in the liquid cladding that homogenously covers the sensor WG. To this end, we first calculate the length-related sensitivity of our Si_3_N_4_ WG for such homogeneous sensing experiments, which amounts to *S*_hom_/*L* = 1450 rad RIU^−1^ mm^−1^. While this value compares well to the value of 1620 rad RIU^−1^ mm^−1^ reported in other sensing experiments with Si_3_N_4_ waveguides^31^, further improvements can be achieved by using advanced designs of the sensor WG, exploiting, e.g. slot structures or subwavelength gratings (SWGs)^32,33^. Using again the standard deviation of the phase measurement, *σ*_Δ*ϕ*_ = 0.2 rad, we determine a limit of detection of LoD_hom_ = 2.29 × 10^−4^ RIU, which may now be compared to that of other demonstrations of WG-based sensors. For meaningful benchmarking, we focus our comparison on experiments that also rely on Si_3_N_4_-based MZI sensors operated at visible wavelengths up to approximately 850 nm. In this wavelength range, there are several reports on actual sensing experiments^29,31,34,35^, among which ref. ^35^ reports a detection limit of LoD_hom_ = 7 × 10^−6^ RIU. This is 30-fold better than our result. Note, however, that this detection limit was achieved with a sensing WG of length L = 15 mm, which is more than eight-fold larger than the 1.8-mm length applied in our example. In addition, the experiment reported in ref. ^35^ relied on a benchtop-type helium-neon (HeNe) laser with a typical output power in the milliwatt range. This is more than four orders of magnitude higher than the average power of 40 nW estimated for our SiNOH lasers based on the 100-mW on-chip pulse peak power and a duty cycle of 4 × 10^−7^. There is hence much room for further improvement of the limit of detection, e.g. by increasing the pulse repetition frequency and hence the output power of the SiNOH laser, see subsequent section. In addition, further improvements of the sensor are possible, by mitigating, e.g., the impact of thermal drift by using reference and sensor WGs that are perfectly matched with respect to their thermal characteristics. Note that the propagation losses of our current Si_3_N_4_ WG are still comparatively high, ranging from 5 to 7 dB cm^−1^, depending on the WG width. Low propagation losses ranging from 0.5 to 2.5 dB cm^−1^, as reported for comparable WGs^8^, could lead to further improvements in the performance of both the laser and the sensor. Based on these estimations, we believe that the performance of visible-wavelength SiNOH-driven MZI sensors could be enhanced to match that of benchtop-type laboratory systems.

### Towards compact portable sensor systems for point-of-care applications

An essential part of the sensor system that requires further elaboration for technically viable point-of-care operation is the pump source. Our current demonstration relies on a bulky benchtop-type Nd:YLF laser, emitting pulses with an energy of 500 nJ at a rather low repetition rate of 20 Hz. The pulse length amounts to 20 ns, leading to a low duty cycle of 4 × 10^−7^ and an accordingly low average output power, which requires a long integration interval of 2 s for the read-out camera. In a point-of-care system, this bulky solid-state laser may be replaced by a compact high-power laser diode, emitting at 520 nm with a CW output power of, e.g. 900 mW^36,37^. Under pulsed operation, these diodes could provide pump-pulse energies typically ranging from 120 to 130 nJ, which is still above the lasing threshold from 30 to 40 nJ of the current devices. Moreover, the repetition rates can be greatly increased to, e.g. to 1 kHz, such that the system could be operated at ~10 times higher optical powers and hence greatly improved signal-to-noise power ratios (SNRs). Note that a repetition rate of 1 kHz is still low enough to allow for relaxation of excited triplet-state dye molecules with typical lifetimes on the order of tens of microseconds^38^ between subsequent pulses. It should also be noted that the design of the laser cavity and the pumping scheme could be further optimized, thereby offering even higher output powers and lower thresholds, which might eventually be compatible with pump-power levels offered by light-emitting diodes (LEDs). On the receiver side, signal read-out and data analysis may rely on compact highly sensitive cameras for visible wavelengths and on compact powerful processors, both of which are readily available on the market.

Regarding the degradation of the laser dye, the current lifetime of at least two hours permits extended measurements even at high-power levels. In the case of slow binding processes, the sensor does not have to be continuously operated as was done in our current experiment but may be periodically turned on and off to further increase the lifetime. This operation mode also leads to reduced power consumption and might allow for battery operation of the entire sensor system. Moreover, the lifetime of organic laser dyes may be greatly improved by hermetic encapsulation layers^39^, possibly in combination with oxygen quenchers^40^.

Another important aspect is the sample preparation and functionalization of the WG surface to enable specific binding of target molecules. Ideally, the system could be operated without any additional sample pretreatment. Massively parallel detection through an array of differently functionalized devices in combination with advanced data analysis might help to improve the specificity of the sensor. Detection of target molecules without pretreatment has, e.g. been demonstrated with saliva^41^ and urine^42^. For complex analyte solutions such as blood, where pretreatment is hitherto unavoidable, compact equipment for point-of-care applications such as portable centrifuges has been demonstrated^43^ and commercialized^44^ in recent years.

### Summary

We demonstrate an integrated sensor system with on-chip SiNOH lasers on the Si_3_N_4_ platform. The sensor is operated by optically pumping the SiNOH lasers from the top with relaxed alignment precision and by detecting the sensor signal with a CCD camera. The whole chip can be fabricated in a single lithography step, and the gain medium of the SiNOH laser can be easily deposited by wafer-level dispensing or printing processes. In a proof-of-concept demonstration, we used this sensor system to detect different concentrations of fibrinogen dissolved in a phosphate-buffered saline solution (PBS). We determined a sensor-length-related sensitivity of *S* = 0.16 rad nM^−1^ mm^−1^.

To our knowledge, this is the first demonstration of an integrated optical circuit driven by a co-integrated low-cost organic light source. We expect that the versatility of the device concept, the simple operating principle, and the compatibility with cost-efficient mass fabrication will make integrated sensors containing SiNOH lasers a highly attractive option for applications in biophotonics and point-of-care diagnostics.

## Materials and methods

### Fabrication of the Si_3_N_4_ waveguides and SiNOH lasers

The Si_3_N_4_ WGs of both the SiNOH laser and sensor circuit are jointly fabricated in a single lithographic step. The WG cores are structured in a 200-nm-thick Si_3_N_4_ layer on top of a 2-µm-thick silicon dioxide layer mechanically supported by a silicon wafer. A negative-tone resist structured via electron-beam lithography and spray development is used as a mask for dry etching of the Si_3_N_4_ layer with a mixture of SF_6_ and CHF_3_. An oxygen plasma etching step is applied to remove the etch mask. After structuring the WG cores, a negative-tone photoresist mrX (mr-X2-P2-XP, Micro Resist Technology, Berlin, Germany) is spin coated and exposed by e-beam lithography to act as a cladding that covers the optical WG except for the laser cavity and the arms of the MZI. To form the light-emitting cladding, laser dye PM597 (Pyrromethene 597, Radiant Dyes Laser & Accessories GmbH, Wermelskirchen, Germany) is dissolved in PMMA at a concentration of 25 μmol/g and then deposited onto the laser WG (Fig. 2a, the magenta-coloured region). In a final step, the reference WG of the MZI is passivated by covering it with glue (MyPolymer MY-136, Ness Ziona, Israel), which is approximately index-matched to water, see Fig. 2c. Phosphate-buffered saline solution (PBS) is used as a solvent for the analyte in the sensing arm.

## Supplementary information

Supplementary File
