# Supplementary material for: Biophotonic sensors with integrated Si3N4-organic hybrid (SiNOH) lasers for point-of-care diagnostics
Source: Light Sci Appl. 2021 Mar 26;10:64. doi: 10.1038/s41377-021-00486-w (PMC7994412; doi:10.1038/s41377-021-00486-w)
Supplement: Supplementary file 1 — Supplementary File [file 41377_2021_486_MOESM1_ESM.docx]

**Supplementary Information for**

**Bio-photonic sensors with integrated Si_3_N_4_-organic hybrid (SiNOH) lasers for point-of-care diagnostics**

Daria Kohler^1,2^, Gregor Schindler^2^, Lothar Hahn^2^, Johannes Milvich^1,4^,

Andreas Hofmann^3^, Kerstin Länge^2^, Wolfgang Freude^1^ and Christian Koos^1,2,*^

^1^ Institute of Photonics and Quantum Electronics (IPQ), Karlsruhe Institute of Technology (KIT), Engesserstrasse 5, 76131 Karlsruhe, Germany

^2^ Institute of Microstructure Technology (IMT), Karlsruhe Institute of Technology (KIT), Hermann-von-Helmholtz-Platz 1, 76344 Eggenstein-Leopoldshafen, Germany

^3^ Institute for Automation and Applied Informatics (IAI), Karlsruhe Institute of Technology (KIT), Hermann-von-Helmholtz-Platz 1, 76344 Eggenstein-Leopoldshafen, Germany

^4^ Robert Bosch GmbH, Robert-Bosch-Campus 1, 71272 Renningen, Germany

* Correspondence and requests for materials should be addressed to C. K. (phone: +49 721 608-42491, email: [christian.koos@kit.edu](mailto:christian.koos@kit.edu))

Supplementary Note 1: Further details on the SiNOH laser

The concept of our SiNOH lasers has previously been reported in a dedicated publication^1^. In this section, we provide further details of the SiNOH spiral laser, focusing on its operating principle, its characterization, and the interpretation of the results.

In the main manuscript, we state that broadband optical feedback is provided from the inner end of the spiral by reflection from the open waveguide (WG) end in the center, possibly in combination with roughness-induced backscattering, whereas coupling of light between neighboring windings of the WG spiral does not play a significant role. This notion is based on numerical investigations of the device. Since a simulation of the full spiral would require prohibitive computational resources, we simplified the structure and checked the coupling between two straight WG with the same dimensions (width *w* = 500 nm, height *h* = 200 nm) and spacing (1.5 µm) as the spiral WG. We determined the propagation constant $\beta_{\text{s}}$ of the symmetric and the antisymmetric modes $\beta_{\text{a}}$ and calculated the coupling length by $\Lambda=\text{π}/\left|\beta_{\text{s}}-\beta_{\text{a}}\right|$. In this definition, $\Lambda$denotes the propagation length after which the power is completely transferred from one WG to the other. For the fundamental quasi-TE and the fundamental quasi-TM modes, Λ is more than 7 m, and we can therefore exclude that coupling of fundamental modes between neighboring windings of the spiral plays a significant role. We repeated the simulation for the next higher-order TE and TM modes, leading to Λ = 20 mm for quasi-TE and Λ = 5 mm for quasi-TM polarization. This is comparable to the length of the spiral, which amounts to 18 mm, such that coupling between higher-order modes might occur. Still, we expect that there will be no significant impact on the lasing performance, since the multimode Si_3_N_4_ WG in the spiral is tapered down to a single-mode WG towards the ring resonator at the output. This taper will strip all higher-order modes such that they will not see any relevant feedback at this side of the resonator. In addition, coupling of higher-order modes in the WG towards the center of the spiral would still be in the forward direction and should thus not lead to enhanced feedback. Note that we use an open spiral in our experiments, rather than a coiled-up loop, where coupling of parallel WG could indeed lead to a frequency-selective feedback to the backward direction, see discussion in our previously published paper^1^.

Based on these results, we expect that optical feedback from the spiral will be predominantly caused by scattering at the rough WG sidewalls, and by reflection from the open inner end of the spiral WG. This feedback will be rather broadband – potentially with some frequency variations due to Anderson localization^2^. In contrast to this, roughness-induced backward scattering in the single-mode ring-resonator is resonantly enhanced by constructive interference of light that scattered back in subsequent round-trips of the ring, thereby building up a counter-propagating resonant mode. This build-up will only end when an equilibrium state is reached, in which the net power loss from the forward-propagation resonator mode to the outside of the resonator and to its backward-propagating counterpart is exactly compensated by the power coupled in from the outside. Due to this resonant enhancement of backscattering, the feedback of the ring is spectrally narrowband and concentrated to the discrete ring resonances. This effect, which has also been reported for other Si_3_N_4_ ring resonators^3^, can be nicely observed by investigating the power emission from GC1 and GC in Fig. 3 of the main manuscript.

According to this model, a spiral WG without a ring resonator should also be able to lase, but the threshold should increase strongly, and the emission should not be concentrated to discrete resonance frequencies that are defined by the ring in our current device. We investigated this aspect experimentally by measuring the lasing characteristics of a spiral that is not coupled to a ring resonator. Instead, the straight orange-colored WG portion in Fig. 3a is directly routed to the chip edge, where we would expect broadband Fresnel reflection at the facet with a rather low power reflection factor of a few percent. For this spiral, we found rather high threshold pump energies in the range of $2...5$μJ– much higher than the 30-40 nJ found for the devices in the main manuscript – along with an unstable spectrum that changes continuously on a time scale of a few seconds. We attribute these variations to thermal drift on the chip, which strongly impacts the emission frequencies associated with Anderson localization. Note that the drastic increase in pump threshold for the device without ring resonator cannot be explained by the reduced feedback alone, but may also result from strong gain competition due to the absence of a frequency-selective feedback. This is in good agreement with previous observations from SiNOH lasers, see Section 4.2 in Ref. 1.

Regarding the fabrication of SiNOH structures, one important aspect is the potential formation of voids or air bubbles in the organic gain medium. We have explored this aspect in the context of our previous publication, where we investigated cross sections of cleaved SiNOH WG, see, e.g., Fig. 3b of Ref. 1. In these investigations, we did not find any indication of voids or air bubbles. This is in good agreement with our experience from silicon-organic hybrid (SOH) devices^4^, where void formation was never observed, even when cladding slot WG with slot widths of the order of 100 nm^5^. We also measured the propagation loss of the Si_3_N_4_ WG in the SiNOH cavity, which, for an un-doped PMMA cladding, amounts to approximately 5 dB/cm for the 500 nm-wide multi-mode spiral WG and to around 7 dB/cm found for narrower 300 nm-wide single-mode WG. We attribute these losses to rough WG sidewalls, see Inset 2 of Fig. 2b of the main manuscript, which may arise from discrete writing patterns of the electron-beam lithography system used for defining the WG. This roughness may also be the origin of resonantly enhanced scattering-induced coupling of light between counter propagating modes in the reflector ring.

We also performed investigations of the SiNOH-laser emission characteristics. Specifically, we simultaneously measured the input-output-power characteristics for the two grating couplers GC1 and GC2, see Fig. S1. We find that the power of the “forward” or clockwise propagating mode in the small ring, probed through GC2, was three times as strong as the power of the “backward” or counter-clockwise propagating mode, probed through GC1, while the threshold observed from the two outputs is essentially the same. This supports the notion that the emission at GC1 results from resonant coupling of counter-propagating modes in the ring. We also investigated the spectrum taken from GC 2 in more detail and extracted the lasing thresholds from the three separate modes individually, Fig. S2. We find that the modes exhibit distinct thresholds with no indication of gain competition. This is ensured by the fact that we chose the FSR of the reflector ring slightly larger than the bandwidth of homogeneous line broadening, that was measured in a previous publication^1^, see Section “On-Chip-SiNOH laser” of the main manuscript.

Figure S2.  Lasing characteristics of different spectral modes. Normalized laser output power of the three dominant laser lines shown in Fig. 3c of the main manuscript as functions of the pump-pulse energy *W*. We find lasing thresholds *W*_th_  of  25 nJ, 43 nJ, and 52 nJ with no indicating of gain competition. This is consistent with the fact that we chose the FSR of the reflector ring slightly larger than the bandwidth of homogeneous line broadening, that was measured in a previous publication.


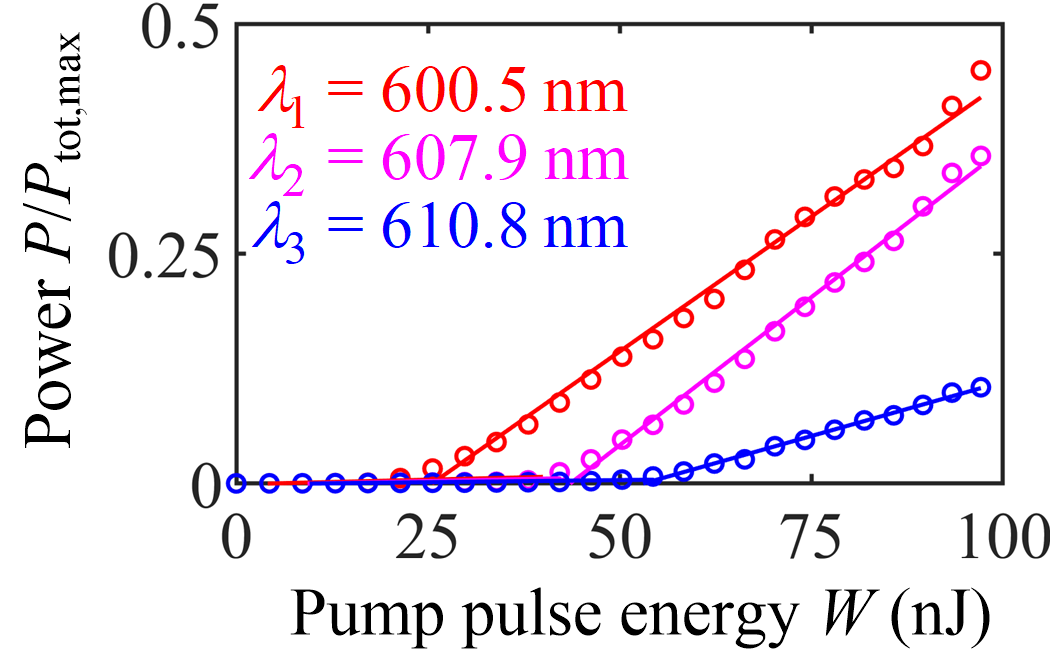


Figure S1.  Laser characteristics extracted from clockwise and counter-clockwise propagating modes in the reflector ring. Normalized laser output powers probed from the counter-clockwise propagating mode through GC1, blue, and from the clockwise-propagating mode through GC 2, red, both as function of the pump-pulse energy *W*. We find essentially the same lasing thresholds, thereby confirming the notion that that the clockwise and the counter-clockwise propagating modes in the reflector ring resonator are resonantly coupled to each other.


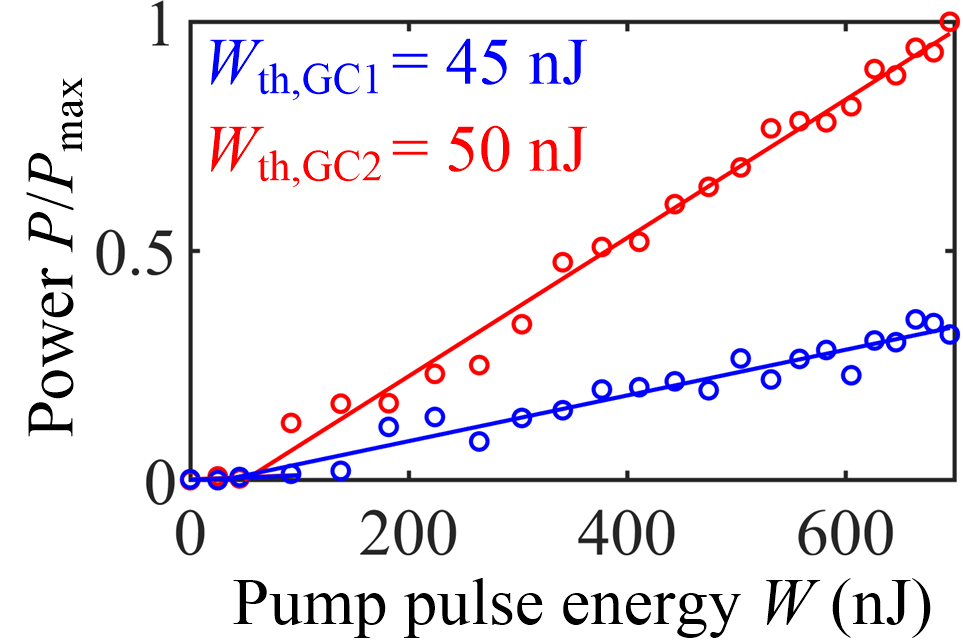


Supplementary Note 2: Monochromatic source

For the analysis of monochromatic laser sources, we assume excitation of the sensor at an angular frequency $\omega$ and a normalized electric field $\underline{E}=\underline{\hat{E}}\exp\left[\mathrm{j}\left(\omega t-\beta z\right)\right]$ having a complex amplitude $\underline{\hat{E}}$ and a propagation constant $\beta$ in the $z$-direction. We represent the optical power as the squared magnitude of the electric field, $P_{\text{source}}=\left|\hat{\underline{E}}\right|^{2}$. The electric field is split by the 2 × 2 MMI into equal portions in the sensing and the reference WG, whereby the field in the reference arm is phase-shifted by $-\pi/2$ with respect to the field in the sensing arm. Reference and sensing arms have equal geometrical arm lengths *L*. Because the reference arm is optically isolated from the analyte by a thick passivation layer, its refractive index $n_{\text{e,R}}$ is constant during sensing experiments. With the vacuum speed of light *c*, the field $\underline{E}_{\text{R}}$ at the end of the reference arm and the associated phase shift can be written as

In the sensing arm, the effective refractive index $n_{\text{e,S}}$ is changed by $\Delta n_{\text{e,S}}$ when molecules attach to the WG surface. This leads to an additional phase shift of compared to the phase without attached molecules,

Combination of the fields by the 3 × 3 MMI leads to a superposition of $\underline{E}_{\text{R}}$ and $\underline{E}_{\text{S}}$, where the field contributions from the sensing arm at the three MMI output ports are phase-shifted by $2\pi/3$ (120°)^6^,

$$\begin{matrix} P_{\text{A}}=\left(\frac{1}{3}\right)\left|\underline{E}_{\text{R}}+\underline{E}_{\text{S}}\exp\left(+\text{j}\frac{2\pi}{3}\right)\right|^{2}, \\ P_{\text{B}}=\left(\frac{1}{3}\right)\left|\underline{E}_{\text{R}}+\underline{E}_{\text{S}}\right|^{2}, \\ P_{\text{C}}=\left(\frac{1}{3}\right)\left|\underline{E}_{\text{R}}+\underline{E}_{\text{S}}\exp\left(-\text{j}\frac{2\pi}{3}\right)\right|^{2}. \end{matrix}$$

The three output powers are detected, analog-to-digital converted, and numerically processed by applying a Clarke transform. For a monochromatic light source, this leads to a complex correlation function (Clarke “field”) $\underline{s}_{\text{mono}}$^7,8^,

When changing $n_{\text{e,S}}$, the Clarke field $s_{\text{mono}}$ describes a circle with radius $\left|\underline{s}_{\text{mono}}\right|=\left|\underline{\hat{E}}\right|^{2}$ in the complex plane. The phase shift changes with $\Delta n_{\text{e,S}}$ and is found by unwrapping the Clarke phase $\arg\left(\underline{s}_{\text{mono}}\right)$,

.

For all values and therefore for all index changes$\Delta n_{\text{e,S}}$, the sensitivity $S_{\text{Clarke}}$is constant and corresponds to $\left|\underline{s}_{\text{mono}}\right|$,

Supplementary Note 3: Polychromatic source

To better reflect the experimental situation of an MZI-based sensor driven by a SiNOH laser, we also investigate the sensor behavior for a polychromatic source with $m=1,2,\ldots,M$ lines at angular frequencies $\omega_{m}$ and real constant amplitudes $\underline{\hat{E}}_{m}$. Each line is represented by an expression of the form $\underline{E}_{m}=\underline{\hat{E}}_{m}\exp\left[\mathrm{j}\left(\omega_{m} t-\beta_{m}z\right)\right]$. The field at the input of MMI_2_, Fig. 2a of the main manuscript, is then $\underline{E}=\sum_{m=1}^{M} \underline{E}_{m}$. The total power as measured with a photodetector results from an average over the observation time *T*, which is large compared to the oscillation period of any two lines in the spectrum, $T\gg2\pi/\left|\omega_{m}-\omega_{n}\right|$ for $m\neq n$. We again represent the optical power of the source as the sum of the squared electric-field magnitudes of the various components,

$$\begin{matrix} P_{\text{source}}=\left\langle\left|E\right|^{2}\right\rangle_{T}=\left\langle\sum_{m=1,n=1}^{M} \underline{E}_{m}\underline{E}_{n}^{*}\right\rangle_{T} \\ =\left\langle\sum_{m=1,n=1}^{M} \hat{\underline{E}}_{m}\underline{\hat{E}}_{n}\exp\left[\mathrm{j}\left(\omega_{m}-\omega_{n}\right)t-\mathrm{j}\left( \beta_{m}-\beta_{n}\right)z\right]\right\rangle_{T} \\ =\sum_{m=1}^{M} \left|\underline{\hat{E}}_{m}\right|^{2} \end{matrix}$$

In these relations, indicates a time average over time *T*. For the analysis of the sensor behavior, we first consider the reference arm. After propagating through the geometrical reference arm length *L*, the spectral components of the field are phase shifted by , and the total field at the end of the reference arm amounts to

For the sensing arm, we assume the same geometrical length *L*. In addition, we account for the changes of the effective refractive indices $n_{\text{e,S,}m}$ by $\Delta n_{\text{e,S,}m}$, which are caused by adsorption of molecules to the WG surface. This leads to an additional phase shift , such that the total field at the end of the sensing arm can be written as

At the output ports of the 3 × 3 MMI, the fields from the reference arm, Eq. (S8), and from the sensing arm, Eq. (S9), are combined in analogy to Eq. (S3). The measured output powers $P_{\text{A}}$, $P_{\text{B}}$and $P_{\text{C}}$ result from an average in analogy to Eq. (S7). Using Eq. (S4), we can derive the Clarke field for a polychromatic source,

where the phase differences of the various spectral components in the sensor and the reference arm depend on the respective frequency $\omega_{m}$,

We expand the function of Eq. (S11) in a Taylor series around a center frequency $\omega_{0}$ and keep only the linear term,

As a center frequency $\omega_{0}$, we choose the average of all emission frequencies $\omega_{m=1...M}$, of the polychromatic light source,

$\omega_{0}=\frac{1}{M}\sum_{m=1}^{M} \omega_{m}$.

The derivative in Eq. (S12) can be expressed by the group refractive index $n_{\text{g}}\left(\omega\right)=n+\omega_{0}\left.\left(\mathrm{d}n/\mathrm{d}\omega\right)\right|_{\omega=\omega_{0}}$ at the center frequency $\omega_{0}$. With the group refractive index $n_{\text{e,g,S}}$ and $n_{\text{e,g,R}}$ of the sample and the reference arm, respectively, and the change $\Delta n_{\text{e,g,S}}$ of the group-refractive index due to adsorption of molecules to the surface of the sensor arm, can be written as

.

The quantity $\Delta n_{\text{e,g}}$ is the group refractive index difference between the sensor and the reference arm in the presence of an analyte. Substituting in Eq. (S10), we can re-write the Clarke field for the polychromatic light source,

Equation (14) can be simplified if $\Delta n_{\text{e,g}}\left|\omega_{m}-\omega_{0}\right|L/c\ll1$ holds, i.e., if the frequency offset $\left|\omega_{m}-\omega_{0}\right|$ of the laser lines is significantly smaller than the free spectral range $c/\left(\Delta n_{\text{e,g}}L\right)$ of the unbalanced MZI. We assume that the interferometer is well balanced, $n_{\text{e,g,S}}\approx n_{\text{e,g,R}}$, and that analyte-induced change $\Delta n_{\text{e,g,S}}$ of the group refractive index is small, thus leading to a large free spectral range. In this case, the exponential in the sum of Eq. (S15) is approximately one, and the phase of $\underline{s}_{\text{poly}}$ is determined by only, i.e., the (narrowband) polychromatic source behaves as if it was monochromatic. In this case, Eq. (S15) can then be re-written as

The phase change can be found by unwrapping $s_{\text{poly}}$ as in Eq. (S5),

For a (wideband) polychromatic source, the condition $\Delta n_{\text{e,g}}\left|\omega_{m}-\omega_{0}\right|L/c\ll1$ is not fulfilled, and the magnitude $\left|\underline{s}_{\text{poly}}\right|$ of the Clarke field depends on the amplitudes and on the frequency span of the mono­chromatic lines,

$$\begin{matrix} \left|\underline{s}_{\text{poly}}\right|^{2}=4\left|\underline{E}_{\text{S}}\underline{E}_{\text{R}}^{\text{*}}\right|^{2} \\ =\sum_{m=1,n=1}^{M} \left|\hat{\underline{E}}_{m}\right|^{2}\left|\underline{\hat{E}}_{n}\right|^{2}\mathrm{e}^{ \mathrm{j}\Delta n_{\text{e,g}}\left(\omega_{m}-\omega_{n}\right)\frac{L}{c}} \\ =\sum_{m=1}^{M} \left|\hat{\underline{E}}_{m}\right|^{4} +\sum_{m=2}^{M} \sum_{n=1}^{m-1} \left|\hat{\underline{E}}_{m}\right|^{2}\left|\hat{\underline{E}}_{n}\right|^{2}2\cos\left(\Delta n_{\text{e,g}} \left(\omega_{m}-\omega_{n}\right)\frac{L}{c}\right). \end{matrix}$$

This in contrast to the case of a monochromatic laser line, for which the magnitude $\left|\underline{s}_{\text{mono}}\right|$ of the Clarke field is constant with increasing , Eqs. (S4) and Eq. (S5). Eq. (S18) can be simplified by assuming $M\gg1$ equidistant laser lines in a maximum spectral range $\Delta\omega_{\max}$, and by postulating approximately equal field amplitudes $\hat{\underline{E}}_{m}\approx\underline{\hat{E}}$. The phase change differences $\Delta n_{\text{e,g}}\left(\omega_{m}-\omega_{n}\right)L/c$ can then be written as

$\Delta n_{\text{e,g}}\left(\omega_{m}-\omega_{n}\right)L/c=\Delta n_{\text{e,g}}\frac{\Delta\omega_{\max}}{M}\left(m-n\right)L/c$.

When evaluating the sums in Eq. (S18), we neglect $M \left|\underline{\hat{E}}\right|^{4}$ compared to $M^{2}\left|\underline{\hat{E}}\right|^{4}$ and replace the sums by integrals, $\sum_{m=2}^{M} \sum_{n=1}^{m-1} \left(\cdot\right)=\int_{m=0}^{M} \int_{0}^{m} \left(\cdot\right)\mathrm{d}m\mathrm{d}n$. This leads to

$$\begin{matrix} \left|\underline{s}_{\text{poly}}\right|^{2} =\left|\hat{\underline{E}}\right|^{4}\int_{m=0}^{M} \int_{n=0}^{m} 2\cos\left(\frac{\Delta n_{\text{e,g}}\Delta\omega_{\max}L}{M c}\left(m-n\right)\right)\mathrm{d}n \mathrm{d}m \\ =\left|\hat{\underline{E}}\right|^{4}M^{2}\frac{\sin^{2}\left(\frac{\Delta n_{\text{e,g}}L}{2c}\Delta\omega_{\max}\right)}{\left(\frac{\Delta n_{\text{e,g}}L}{2c}\Delta\omega_{\max}\right)^{2}} \\ =P_{\text{source}}^{2} \frac{\sin^{2}\left(\frac{\Delta n_{\text{e,g}}L}{2c}\Delta\omega_{\max}\right)}{\left(\frac{\Delta n_{\text{e,g}}L}{2c}\Delta\omega_{\max}\right)^{2}}. \end{matrix}$$

Equation (S20) illustrates that the magnitude $\left|\underline{s}_{\text{poly}}\right|$ decreases for increasing $\Delta n_{\text{e,g}}$ and therefore for increasing $\Delta n_{\text{e,g,S}}$. For , i.e., $\Delta\omega_{\text{max}}\to0$, the result for one monochromatic line is reproduced, Eq. (S4).

Supplementary Note 4: 3 × 3 MMI, Clarke field transformation and resolution

In our experiments, we rely on MZI with 3 × 3 MMI as power combiners at the output. A key advantage of this approach is that phase ambiguities within a 2π-interval can be avoided. This is possible due to the detection of three sensor signals that have a fixed phase difference of 2π/3 (120°) with respect to each other. The transformation of these signals to a Clarke field, leads to a locus in form of a circle in the complex plane, where the position on the curve is well defined within a phase shift of 2π, see Eq. (S4) and Eq. (S15). In contrast to that, MZI with 2 × 2 MMI only deliver two perfectly complementary signals at the output. In these devices, any phase difference leads to exactly the same output powers as its 2π -complement . As a consequence, unambiguous phase extraction is possible only within intervals of π.

To illustrate the locus-correction of the complex Clarke field $\underline{s}$ used in the main manuscript, we compare the temporal evolution of the raw, i.e., uncorrected Clarke field to its locus-corrected counterpart, Fig. S3a. The underlying data was extracted from the binding experiment of 121 nM fibrinogen in phosphate-buffered saline solution (PBS), see right-hand part of the binding curve in Fig. 6a of the main manuscript. In our experiments, the MMI couplers were already optimized for uniform power splitting ratios and phase shifts, such that the impact of the locus correction is rather small. As a consequence, the binding curves extracted from the corrected and the un-corrected data are essentially identical, see Fig. S3b. The appreciable fluctuations of the Clarke fields in our measurements originate from rather low signal-to-noise power ratios (SNR) of the recorded signals. This can be improved by higher output powers of the SiNOH lasers, which can be achieved by using higher pulse repetition frequencies, see Section “Towards compact portable sensor systems for point-of-care applications” in the main manuscript. For the current sensor implementation, we quantify the variations of the phase shift extracted while flushing the sensor with PBS, leading to a standard deviation of .

Supplementary references

Figure S3. Exemplary Clarke-field correction and binding curve.  a Evolution of the uncorrected (blue) and locus-corrected (green) Clarke field $\underline{s}$ in the complex $\underline{s}$-plane for the binding experiment of 121 nM fibrinogen, see right-hand part of the binding curve in Fig. 6a of the main manuscript. b Measured phase shift as a function of time for the uncorrected (blue) and the corrected (green) binding curve. The encircled numbers in red and black mark subsequent phases as they are indicated in Subfigure S3a. Inset: Magnified portion of the corrected data (green) that were detected during flushing the sensor with phosphate buffered saline solution (PBS). The standard deviation of the binding curve amounts to .


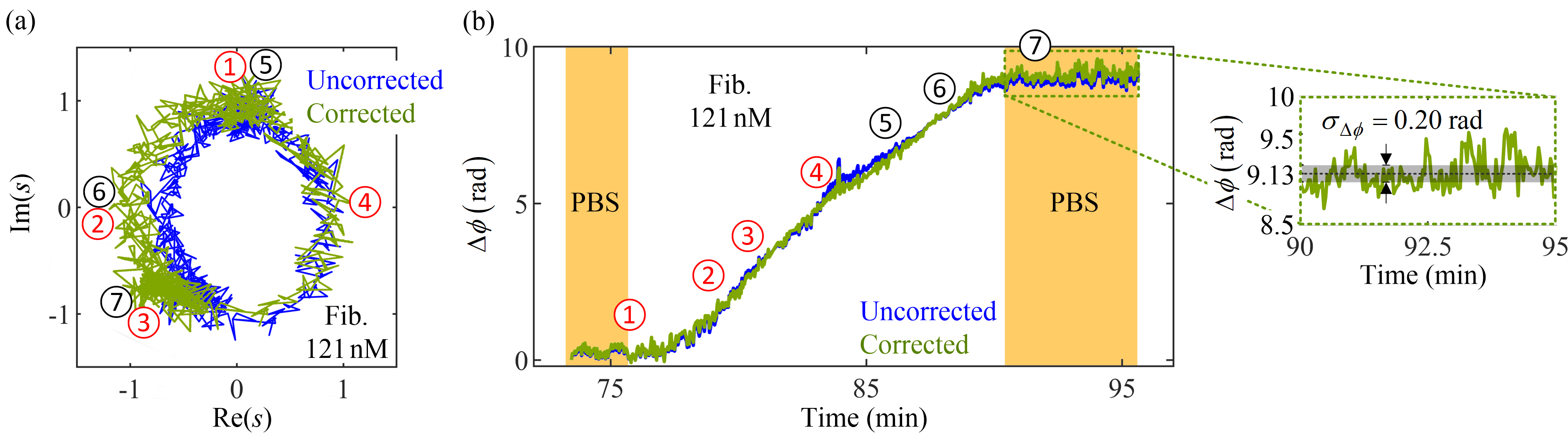


1. Kohler, D. *et al.* Lasing in Si_3_N_4_-organic hybrid (SiNOH) waveguides. *Optics Express* **28**, 5085-5104 (2020).
2. Anderson, P. W. Absence of diffusion in certain random lattices. *Physical Review* **109**, 1492–1505 (1985).
3. Raja, A. S. *et al.* Electrically pumped photonic integrated soliton microcomb. *Nature Communications* **10**, 680 (2019).
4. Kieninger, C. *et al.* Ultra-high electro-optic activity demonstrated in a silicon-organic hybrid modulator. *Optica* **5**, 739-748 (2018).
5. Koeber, S. *et al.* Femtojoule electro-optic modulation using a silicon–organic hybrid device. *Light: Science & Applications* **4**, e255 (2015).
6. Bachmann, M., Besse, P. A. & Melchiorm, H. General self-imaging properties in N × N multimode interference couplers including phase relations. *Applied Optics* **33**, 3905-3911 (1994).
7. Halir, R. *et al.* Direct and sensitive phase readout for integrated waveguide sensors. *IEEE Photonics Journal* **5**, 6800906 (2013).
8. Reyes-Iglesias *et al.* High-performance monolithically integrated 120° downconverter with relaxed hardware constraints. *Optics Express* **20**, 5725 (2012).
